# Extract of *Gardenia jasminoides* Ellis Attenuates High-Fat Diet-Induced Glycolipid Metabolism Disorder in Rats by Targeting Gut Microbiota and TLR4/Myd88/NF-κB Pathway

**DOI:** 10.3390/antiox13030293

**Published:** 2024-02-28

**Authors:** Chenghao Lv, Xin Liu, Shiyun Chen, Yuhang Yi, Xinnian Wen, Tao Li, Si Qin

**Affiliations:** 1 College of Bioscience and Biotechnology, Hunan Agricultural University, Changsha 410128, China; lvchenghao@stu.hunau.edu.cn (C.L.); yu_xin@stu.hunau.edu.cn (X.L.); 2 College of Food Science and Technology, Hunan Agricultural University, Changsha 410128, China; shiyunchen0531@stu.hunau.edu.cn (S.C.); yiyuhang@stu.hunau.edu.cn (Y.Y.); wenxinnian@stu.hunau.edu.cn (X.W.); 3 Hunan Agricultural Product Processing Institute, Hunan Academy of Agricultural Sciences, Changsha 410125, China

**Keywords:** crocin, *Gardenia jasminoides* Ellis, glycolipid metabolism disorder, gut microbiota, TLR4/Myd88/NF-κB pathway

## Abstract

*Gardenia jasminoides* Ellis is abundant in crocin and has a longstanding historical usage both as a dietary and natural ethnic medicine. Enhanced studies have increasingly revealed the intricate interplay between glycolipid metabolism and gut microbiota, wherein their imbalance is regarded as a pivotal indicator of metabolic disorders. Currently, the precise molecular mechanism of the crude extract of crocin from *Gardenia jasminoides* Ellis (GC) targeting gut microbiota to regulate glycolipid metabolism disorder is still unclear. Firstly, we explored the effect of GC on digestive enzymes (α-amylase and α-glucosidase) in vitro. Secondly, we investigated the effect of GC on the physical and chemical parameters of high-fat diet (HFD) rats, such as body weight change, fasting blood glucose and lipid levels, and liver oxidative stress and injury. Then, 16S rDNA sequencing was used to analyze the effects of GC on the composition and structure of gut microbiota. Finally, the impact of GC on the TLR4/Myd88/NF-κB signaling pathway in the intestine was assessed by Western Blotting. In the present study, GC was found to exhibit a hypoglycemic effect in vitro, by inhibition of digestive enzymes. In animal experiments, we observed that GC significantly reduced fasting blood glucose, TC, and TG levels while increasing HDL-C levels. Additionally, GC demonstrated hepatoprotective properties by enhancing liver antioxidative capacity through the upregulation of SOD, CAT, and GSH-Px, while reducing ROS. 16S rDNA sequencing results showed that GC had a significant effect on the gut microbiota of HFD rats, mainly by reducing the ratio of *Firmicutes*/*Bateroidota*, and significantly affected the genera related to glycolipid metabolism, such as *Akkermansia*, *Ligilactobacillus*, *Lactobacillus*, *Bacteroides*, *Prevotellaceae*, etc. The Western Blotting results demonstrated that GC effectively downregulated the protein expressions of TLR4, Myd88, and NF-κB in the intestine of HFD rats, indicating that GC could target the TLR4/Myd88/NF-κB pathway to interfere with glycolipid metabolism disorder. Correlation analysis revealed that GC could target the *Akkermansia*-TLR4/Myd88/NF-κB pathway axis which attenuates glycolipid metabolism disorder. Therefore, this study establishes the foundation for GC as a novel therapeutic agent for glycolipid metabolism disorder chemoprevention, and it introduces a novel methodology for harnessing the potential of natural botanical extracts in the prevention and treatment of metabolic syndrome.

## 1. Introduction

Glycolipid metabolism disorder, as a crucial characteristic of metabolic diseases, poses a significant threat to human health. Glycolipid metabolism disorder is characterized by dysregulation in glucose and lipid metabolism, accompanied by insulin resistance, oxidative stress, inflammatory response, and an imbalance in intestinal flora as the core pathological mechanisms [1,2,3]. This condition manifests clinically as hyperglycemia, dyslipidemia, non-alcoholic fatty liver disease, obesity, hypertension, and atherosclerosis, either individually or in combination. Therefore, a comprehensive understanding and integrated approach to prevention and control are imperative. In the context of glucose and lipid metabolic disorders, insulin resistance (IR) can perpetuate itself through a negative feedback loop, leading to hyperinsulinemia and hyperglycemia [4]. Within the state of IR, the inhibitory effect of insulin on lipolysis is significantly attenuated, resulting in the substantial release of Free Fatty Acids (FFAs) into systemic circulation [5]. The elevation of FFAs induces an upregulation in the synthesis and secretion of triglycerides (TG) within the liver, consequently leading to an increase in low-density lipoprotein cholesterol (LDL-C) levels and a reduction in high-density lipoprotein cholesterol (HDL-C) levels, ultimately resulting in hypertriglyceridemia. Simultaneously, elevated levels of FFAs induce lipotoxicity, resulting in impaired glucose uptake in peripheral tissues and subsequent IR [6]. These interconnected mechanisms establish a positive feedback loop that accelerates the onset and progression of metabolic disorders related to glycolipid metabolism. The oxidative stress caused by the imbalance of REDOX reaction causes reactive oxygen species (ROS) to increase dramatically in the body, which eventually leads to the occurrence of obesity, diabetes, and other chronic diseases dominated by metabolic syndrome [7]. In addition, more and more experimental studies have proved that intestinal barrier and intestinal flora can directly or indirectly affect oxidative stress, inflammation, and insulin resistance, and then affect glycolipid metabolism disorder [8].

The maintenance of the body’s internal environment relies on the crucial integrity of the intestinal barrier. Gut microbiota influence glucose metabolism through the gut–liver axis [9]. Well-balanced gut microbiota play a crucial role in maintaining the integrity of the intestinal barrier, which serves as a protective shield against harmful substances and pathogens entering the systemic circulation. However, dysbiosis of the microbiota can disrupt this barrier function, allowing bacteria and their metabolites to penetrate into the gut–liver system, thereby triggering systemic inflammation. Obesity induces intestinal permeability and results in elevated circulating levels of lipopolysaccharides (LPS) from the outer membrane of Gram-positive bacteria in the intestine. LPS activates Pattern Recognition Receptors (PRRs), leading to the release of pro-inflammatory cytokines such as TNF-α and IL-1β, which activate TLR 4 receptors and NF-κB pathways, triggering a cascade of inflammatory responses in both the intestines and liver, disrupting the insulin signaling pathway and causing insulin resistance [10]. Due to the intricate regulatory mechanisms governing glycolipid metabolism, the singular target of pharmacotherapy, and the potential for adverse reactions, it poses a formidable challenge to identify safe, efficacious, and comprehensive intervention strategies aimed at ameliorating glycolipid metabolism disorder. The field of Traditional Chinese Medicine (TCM) exhibits distinct characteristics and advantages in the prevention and treatment of glycolipid metabolism disorder, showcasing its multi-targeted, multi-pathway, and multi-mechanistic attributes [11]. 

**Figure 1:**
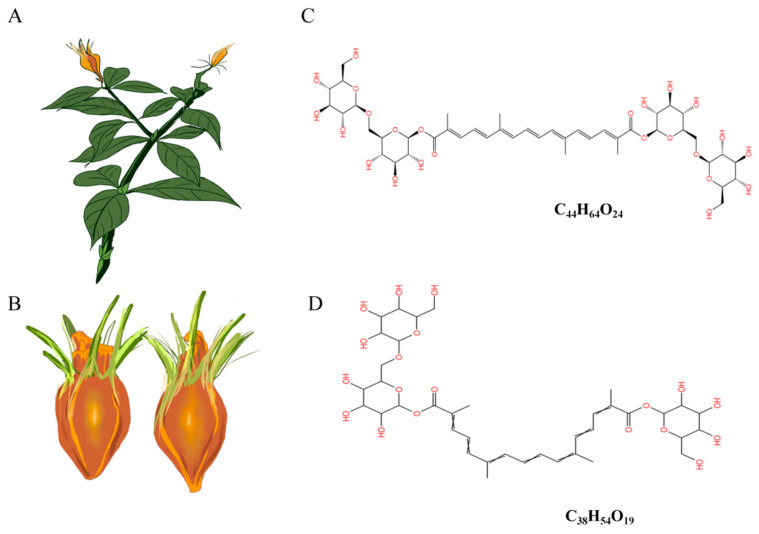
Schematic representation of the plant (**A**) and the mellow fruit (**B**) of *Gardenia jasminoides* Ellis and the molecular structural formula of crocin-1 (**C**) and crocin-2 (**D**).

*Gardenia jasminoides* Ellis (Figure 1A) is a plant with a rich history of traditional ethnic medicine and consumption. The fruit (Figure 1B) of it contains various bioactive components, including crocin, crocetin, iridoid glycosides, volatile oils, terpenes, and organic acids, etc. [12]. Crocin, the primary active compound found in *Gardenia jasminoides* Ellis, is the sole water-soluble carotenoid naturally occurring in nature [13]. It predominantly comprises water-soluble unsaturated fatty acid glucosides such as crocin-1 (Figure 1C) and crocin-2 (Figure 1D). Crocin exhibits pharmacological activities encompassing neuroprotection, anti-tumor effects, antioxidation, anti-thrombotic properties, anti-depressant actions, cardioprotection, immune modulation, amelioration of retinopathy, and regulation of blood lipids [14]. Our previous study found that crocin has hypoglycemic and lipid-lowering effects, but the underlying molecular mechanism by which crocin regulates glycolipid metabolism disorder remains poorly elucidated. Furthermore, it remains unclear whether crocin targets the regulation of intestinal flora to enhance the intestinal barrier function, thereby modulating glycolipid metabolism disorder.

In this study, we employed the crude extract of crocin from *Gardenia jasminoides* Ellis (GC) as the experimental material to investigate the impact of GC on digestive enzymes in vitro, and its effects on blood glucose, blood lipids, and physicochemical indicators of liver oxidative stress in high-fat diet (HFD)-induced rats. Additionally, 16s rDNA sequencing and Western Blotting assays were utilized to explore the influence of GC on gut microbiota and intestinal barrier integrity, particularly focusing on the TLR4/Myd88/NF-κB pathway. We aim to provide theoretical support and establish a foundation for the future application of GC by investigating its potential in attenuating glycolipid metabolism disorders.

## 2. Materials and Methods

### 2.1. Materials and Chemicals

Crude extract of crocin from *Gardenia jasminoides* Ellis (GC) was purchased from Hi-Tech Bio-Agro Co., Ltd. (Yueyang, China). GC powder with 60% purity was obtained by water extraction. Simvastatin (SIM, 99% min) was purchased from Tianjin Huairen Pharmaceutical Co., Ltd. (Tianjin, China). The nutrient composition of the high-fat diet (HFD) was 19.4% crude protein, 60.0% crude fat, and 20.6% carbohydrate, and it was obtained from Trophic Animal Feed High-tech Co., Ltd. (Nantong, China). High-fat diet formulation was provided in Table S1.

### 2.2. Animals and Models

A total of 25 Sprague–Dawley rats (Laboratory animal production License No. SCXK (Xiang) 2019-0004) were randomly allocated into 5 groups: Normal group (fed a normal diet), Control group (fed high-fat diet), SIM group (HFD + Simvastatin), LGC group (high-fat diet + 100 mg/kg GC), and HGC group (high-fat diet + 200 mg/kg GC), with 5 rats in each group. All animals were raised in a barrier environment of the Hunan Provincial Center for Drug Safety Evaluation and Research (License No. SYXK (Xiang) 2020-0015). The rats in each group received daily gavage feeding for 12 weeks. The body weight of the rats was observed and recorded weekly. Feces and intestinal samples from each group were collected aseptically at the 12th week and placed into sterile enzyme-free EP tubes before being cryopreserved at −80 °C.

### 2.3. Serum and Liver Physicochemical Parameters

Every two weeks, blood samples were collected from the posterior orbital venous plexus of the rats in each group, and centrifuged at 3000 rpm for 10 min to obtain serum. Serum levels of LDL-C, TC, TG, and HDL-C were measured using Assay Kits (FUJIFILM Wako Pure Chemical Corporation, Japan). The specific procedures were conducted as follows: blank wells were supplemented with 2.5 μL of distilled water, standard wells were supplemented with 2.5 μL of calibrator, sample wells were supplemented with 2.5 μL of rat serum, all three wells received an addition of 2.5 μL working solution, subsequent shaking and incubation at a temperature of 37 °C for a duration of 10 min took place, and the absorbance was measured at a wavelength of 500 nm. At the 4th, 8th, and 12th week of administration, fasting blood samples of the Normal group, Control group, SIM group, LGC group, and HGC group were collected from the tail tip after a fasting period of 12 h to measure fasting blood glucose levels using blood glucose detection strips and a Blood Glucose Meter (Changsha Sinocare Inc., Changsha, China). The liver tissue weighing 0.1 g was precisely measured, followed by the addition of 1 mL of the specified extraction reagent from the assay kit, and subsequent homogenization in an ice bath. Centrifugation was performed at a relative centrifugal force of 8000× *g* for 10 min at 4 °C. The resulting supernatant was collected to determine the levels of hepatic reactive oxygen species (ROS), superoxide dismutase (SOD), catalase (CAT), glutathione peroxidase (GSH-Px), aspartate aminotransferase (AST), and alanine aminotransferase (ALT) by using Assay Kits (Beijing Solarbio Science & Technology Co., Ltd., Beijing, China) according to the provided instructions. 

### 2.4. Histopathological Analysis

At the conclusion of the 12 weeks, rats from each group were anesthetized and euthanized following exsanguination of the abdominal aorta. A segment of the left lobe liver tissue was excised from each rat and fixed in 4% paraformaldehyde. After 24 h, the liver samples were embedded in paraffin and subsequently sectioned using a cryostat. The sections were then stained with hematoxylin-eosin (H&E) for histological analysis. Finally, pathological imaging was performed using the DFC420C pathological imaging system (Leica, Germany).

### 2.5. 16S rDNA Sequencing

Feces and intestinal tissue samples were collected, and DNA was extracted from the samples using the E.Z.N.A.^®^ Stool DNA Kit (Omega, GA, USA). Nuclear-free water was used as a control in the blank group. The total DNA was eluted in 50 μL of elution buffer and stored at −80 °C. The prokaryotic small subunit (16S rRNA genes V3-V4 region) can be amplified using primers 341f (5′-CCTACGGGGNGGCWGCAG-3′) and 805r (5′-GACTAChVGGGGTATCC-3′). The 5′ end of the primers was labeled with specific barcodes corresponding to each sample, while the universal primers were subjected to sequencing. A total reaction volume of 25 μL was used for PCR amplification, comprising a mixture containing 25 ng template DNA PCR pre-mixture, 2.5 μL of each primer, and PCR-grade water to adjust the final volume. The PCR conditions for amplification of the prokaryotic 16S fragment involved an initial denaturation step at 98 °C for 30 s, followed by 32 cycles of denaturation at 98 °C for 10 s, annealing at 54 °C for 30 s, and extension at 72 °C for 45 s. A final extension was performed at 72 °C for a duration of 10 min. The confirmation of PCR products was achieved through electrophoresis on a gel made with a concentration of agarose gel set to be at least two percent. Throughout the DNA extraction process, ultrapure water was utilized as a substitute for the sample solution in order to eliminate any possibility of false positive PCR results, serving as negative controls. The PCR products were purified using AMPure XP beads (Beckman Coulter Genomics, MA, USA) and quantified with Qubit3.0. Amplification pools were prepared for sequencing, and the size and number of amplified libraries were assessed using an Agilent 2100 Bioanalyzer (Agilent, MA, USA) and an IlluMina library quantification kit, respectively. These libraries were sequenced on a NovaSeq PE250 platform.

### 2.6. Western Blotting

Liver tissue protein was extracted using the total protein extraction kit (Solarbio science and technology Co., Ltd., Beijing, China) following the manufacturer’s instructions, and protein samples were collected by centrifugation at 8000× *g* for 10 min at 4 °C. The supernatant was subsequently collected, and protein concentrations were determined using the BCA Protein Assay Kit (Beyotime Biotechnology Co., Ltd., Shanghai, China). Then, the proteins were separated by 10% SDS-PAGE and transferred onto PVDF membranes (Amersham Pharmacia Biotech, Amersham, UK). The membranes were blocked with 5% skim milk at room temperature for 1 h, followed by overnight incubation at 4 °C with the primary antibody. Primary antibodies against TLR4, MYD88, and NF-κB were obtained from Abcam (Abcam, Waltham, MA, USA) and used at a dilution of 1: 2000; antibodies against β-actin were obtained from Abcam and were used at a dilution of 1:1000. Afterward, they were further incubated for 1 h with an HRP-conjugated secondary antibody. The secondary antibodies were anti-rabbit (Sigma-Aldrich, St. Louis, MI, USA) and were used at a dilution of 1:20,000. Finally, an appropriate amount of ECL chemiluminescence solution was added to the PVDF membrane, which was exposed and developed by Image Quant LAS 4000 mini (General Electric Company, Morrison, CO, USA). The protein bands were quantified by using software such as SmartDraw (6.0) and ImageJ (https://imagej.net/ij/).

### 2.7. Inhibition of Digestive Enzyme Activity

The 1% starch solution was heated to 100 °C and agitated for 10 min to induce gelatinization. A total of 100 μL GC with different concentrations (0, 0.25, 0.5, 0.75, 1.00, 1.25, 1.50, 1.75, 2.00 mg/mL), phosphate buffer (100 mmol/L, pH 6.8), and 50 μL α-amylase (2 U/mL, Shanghai yuanye Bio-Technology Co., Ltd., Shanghai, China) were precultured for 30 min at 37 °C. Subsequently, 100 μL of pre-gelatinized 1% soluble starch was added as the reaction substrate and incubated at 37 °C for 20 min, then 500 μL of DNS chromogenic reagent was added and boiled for 5 min, and finally diluted with 750 μL distilled water. The absorbance values of the mixture were measured at 540 nm using Multiskan SkyHigh ELIASA (Thermo Fisher Scientific Inc., Waltham, MA, USA), and without adding GC as a blank control. The IC50 value was calculated by the regression equation. The inhibition rate of α-amylase is shown in Equation (1):

(1) $$\mathrm{The}\;\mathrm{inhibition}\;\mathrm{rate}\;\mathrm{of}\;\alpha \text{-}\mathrm{amylase}=\frac{A_{\mathrm{control}}-A_{\mathrm{sample}}}{A_{\mathrm{control}}}$$

where A_control_ represents the absorbance at 540 nm without GC added, and $A_{\mathrm{sample}}$ represents the absorbance at 540 nm of different concentrations of GC.

First, 100 μL α-glycosidase enzymes (1.0 U/mL, Shanghai yuanye Bio-Technology Co., Ltd., Shanghai, China) with different concentrations of 50 μL GC (0, 0.25, 0.5, 0.75, 1.00, 1.25, 1.50 mg/mL) were incubated at 37 °C for 10 min. Then, 50 μL of 3.0 mmol/L pNPG was added as the reaction substrate and incubated at 37 °C for 20 min. Finally, the reaction was terminated by adding 1 mL of 0.1 mol/L Na_2_CO_3_. The absorbance value at 405 nm was determined, and α-glucosidase activity was calculated based on the yellow p-nitrophenol released from PNG. The absorbance value at 405 nm was determined, and α-glucosidase activity was calculated based on the yellow p-nitrophenol released from PNG. The IC50 value was calculated by the regression equation. The inhibition rate of α-glycosidase is shown in Equation (2):

(2) $$\mathrm{The}\;\mathrm{inhibition}\;\mathrm{rate}\;\mathrm{of}\;\alpha \text{-}\mathrm{glycosidase}=\frac{A_{\mathrm{control}}-A_{\mathrm{sample}}}{A_{\mathrm{control}}}$$

where A_control_ represents the absorbance at 405 nm without GC added, and $A_{\mathrm{sample}}$ represents the absorbance at 405 nm of different concentrations of GC.

### 2.8. Statistical Analysis

All experiments were conducted in triplicate and the data from each independent experiment were analyzed using SPSS 26.0 software. GraphPad Prism 9 was utilized for graphical representation. Results are presented as standard error of mean (SEM) of the number of experiments performed. Statistical significance was determined by one-way ANOVA with multiple comparisons, where *p* < 0.05 was considered significant.

## 3. Results 

### 3.1. Effect of GC on Activity of α-Amylase and α-Glucosidase In Vitro

**Figure 2:**
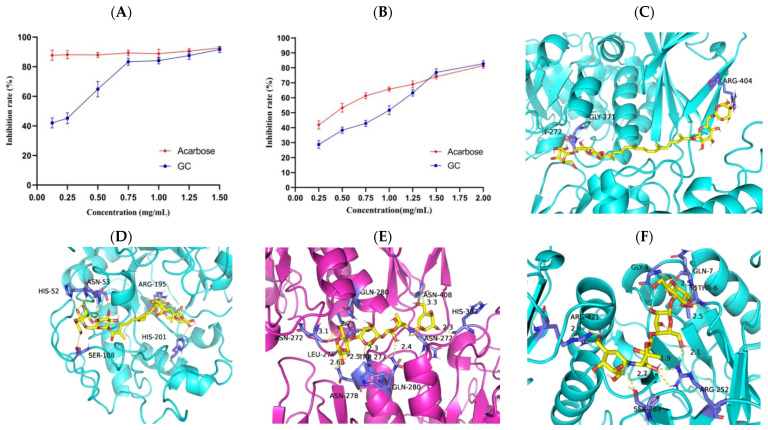
Inhibition rate and binding capacity of GC on α-glucosidase and α-amylase. The impact of GC on the inhibition rate of α-glycosidase (**A**) and α-amylase (**B**). Molecular docking of GC to α-glycosidases (**C**) and α-amylase (**D**). Molecular docking of acarbose to α-glycosidases (**E**) and α-amylase (**F**).

α-amylase and α-glucosidase play important roles in the hydrolysis of polysaccharides, and the inhibition of these two enzymes can effectively reduce postprandial hyperglycemia. To assess the potential of GC in reducing glucose levels, the in vitro digestive enzyme inhibition assay was conducted. The results depicted in Figure 2A,B demonstrate a significant dose-dependent inhibition of α-amylase and α-glucosidase activities by GC. Specifically, the maximum inhibition rate of GC on α-amylase was 82.73 ± 1.99%, which was higher than the maximum inhibition rate of acarbose on α-amylase (81.46 ± 1.77%). The maximum inhibitory effect of GC on α-glucosidase was 91.64 ± 2.06%, which closely resembled the maximum inhibition observed with acarbose (92.67 ± 0.53%) against α-glucosidase. The above results suggest that GC exhibits promising hypoglycemic ability in vitro. The molecular docking results of GC and acarbose with α-glucosidase are presented in Figure 2C and 2E, respectively. The calculated binding affinities for acarbose and GC to α-glucosidase were −9.1 kcal/mol and −9.5 kcal/mol, respectively, indicating a strong affinity between GC and α-glucosidase. The molecular docking results of GC and acarbose with α-amylase are shown in Figure 2D and 2F, respectively. Acarbose and GC have predicted binding affinities of −8.2 kcal/mol and −8.8 kcal/mol to α-amylase, indicating a strong affinity between GC and α-amylase. 

### 3.2. Effect of GC on Physicochemical Parameters Associated with Glycolipid Metabolism in HFD Rats

**Figure 3:**
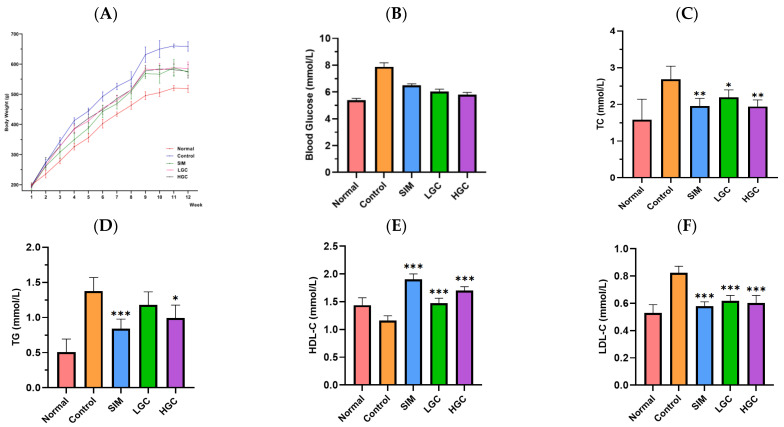
Effect of GC on body weight (**A**), blood glucose (**B**), TC (**C**), TG (**D**), HDL-C (**E**), and LDL-C (**F**) in HFD rats. SIM, LGC, HGC vs. Control, * *p* < 0.05, ** *p* < 0.01, *** *p* < 0.001.

As shown in Figure 3A, with the increase in feeding duration, there was a gradual increase in weight for each group; however, the weight values of the Normal, SIM, LGC, and HGC groups remained consistently lower than that of the Control group. After the 9th week, the body weight of LGC exhibited a consistent trend, while the body weight of the HGC group demonstrated a declining pattern. The impact of GC on rat body weight control was comparable to that of the SIM group and approached that observed in the Normal group. The findings suggest that GC exerts effective control over the body weight of rats.

As shown in Figure 3B–F, compared with the Normal group, the levels of blood glucose, TC, TG, and LDL-C in the Control group were significantly upregulated, indicating that the modeling was successful. In comparison to the Control group, both the LGC and HGC groups exhibited a discernible decline in blood glucose levels. The levels of TC and LDL-C in the LGC group were significantly decreased (*p* < 0.01 or *p* < 0.001), while the levels of HDL-C were significantly increased (*p* < 0.001). The levels of TC, TG, and LDL-C in the HGC group were significantly decreased (*p* < 0.01 or *p* < 0.001), and the levels of HDL-C were significantly increased (*p* < 0.001). The hypolipidemic efficacy of the HGC group surpassed that of the LGC group, while demonstrating comparable effectiveness to the SIM group.

### 3.3. Effect of GC on Liver Injury and Oxidative Stress in HFD Rats

**Figure 4:**
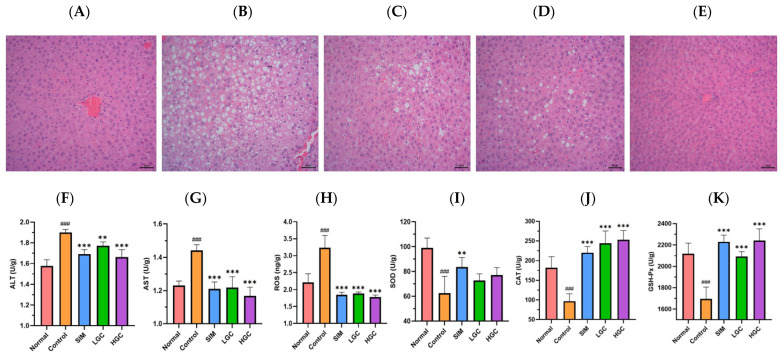
H&E stains of liver tissue in rats, (**A**) Normal, (**B**) Control, (**C**) SIM, (**D**) LGC, (**E**) HGC. Effect of GC on liver ALT(**F**), AST (**G**), ROS (**H**), SOD (**I**), CAT (**J**), GSH-Px (**K**) levels in rats. Control vs. Normal, ^###^ *p* < 0.001. SIM, LGC, HGC vs. Control, ** *p* < 0.01, *** *p* < 0.001.

The H&E staining results of the histological examination are shown in Figure 4. As shown in Figure 4A, the liver tissue cells in the normal group exhibited a well-organized arrangement, displaying typical cell morphology, abundant cytoplasm, and distinct boundaries. The liver of the Control group exhibited a significant increase in both the number of lipid droplets and inflammatory cell infiltration, as depicted in Figure 4B, compared to the Normal group. As depicted in Figure 4D,E, GC could effectively mitigate HFD-induced liver tissue pathological changes, including hepatic steatosis, follicular degeneration, inflammatory cell infiltration, and reduced lipid droplet accumulation. These findings demonstrate the hepatoprotective potential of GC.

Compared to the Normal group, the Control group exhibited a significant increase in levels of ALT, AST, and ROS (*p* < 0.001), while levels of SOD, CAT, and GSH-Px were significantly decreased (*p* < 0.001). Compared with the Control group, the levels of ALT, AST, and ROS in the serum of HFD rats in the LGC and HGC groups were significantly decreased (*p* < 0.01 or *p* < 0.001), the levels of CAT and GSH-Px were significantly increased (*p* < 0.001), and the level of SOD was increased but not significantly. The results showed that GC could improve the liver injury induced by HFD.

### 3.4. Effect of GC on Gut Microbiota in HFD Rats

**Figure 5:**
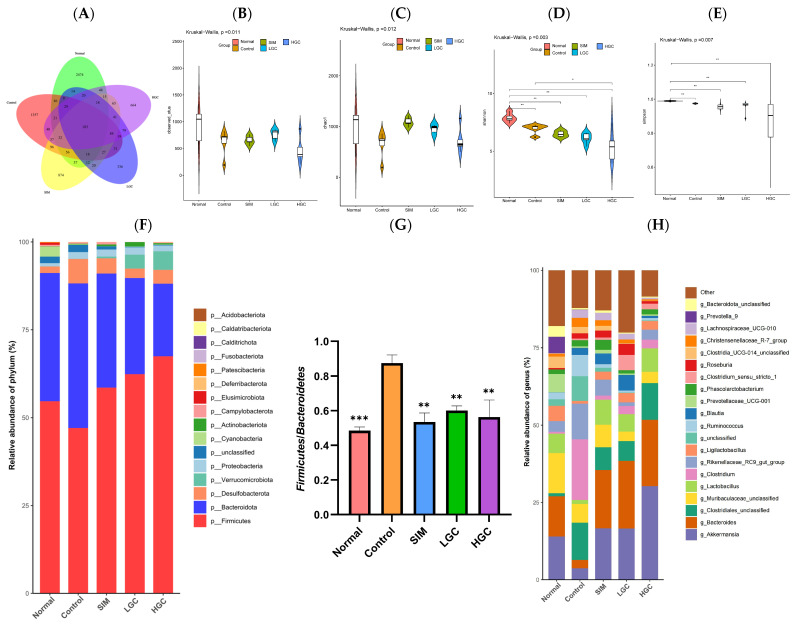

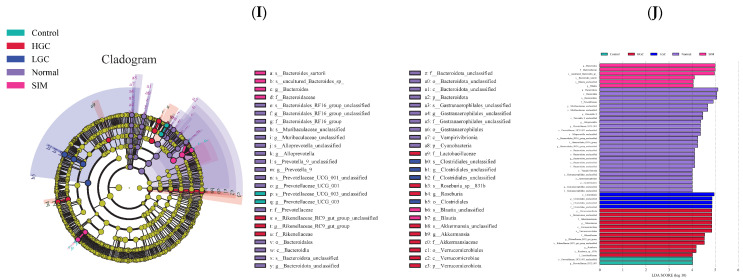
The impact of GC on the diversity and composition of gut microbiota in HFD rats. (**A**) Venn diagram of microflora differences between groups. (**B**) Observed OTU. (**C**) Chao1 index. (**D**) Shannon index. (**E**) Simpson index. (**F**) Phylum level. (**G**) Abundance ratio of *Firmicutes*/*Bacteroidetes*. (**H**) Genus level. (**I**) Taxonomic cladogram obtained from LEfSe analysis. (**J**) LEfSe analysis identified the microbiota phylotypes with the statistical difference in abundance among groups. SIM, LGC, HGC vs. Control, * *p* < 0.05, ** *p* < 0.01, *** *p* < 0.001.

We employed 16s rDNA gene sequencing technology to assess the impact of GC on the diversity and composition of gut microbiota. In Figure 5A, according to the abundance of the obtained eigenvalues, the number of common features in each group is calculated, and the number of common and unique features in each group is visually presented by Venn diagram. The five comparison groups collectively exhibited a total of 183 identical features, with the Normal group demonstrating significantly higher prevalence compared to the other groups. Compared to the Control group, the difference of feature in the Normal group, SIM group, LGC group, and HGC group was 79.58%, 74.92%, 81.18%, and 79.05%, respectively, indicating that the gut microbiota abundance was different in each group. 

The assessment of Alpha diversity primarily relied on the Chao1 index, Observed OTUs, Shannon index, and Simpson index. The Chao1 index and Observed OTUs primarily serve as indicators of species richness in the samples. A higher index value corresponds to a greater number of OTUs, suggesting a larger diversity of species within the sample. As shown in Figure 5B,C, the Chao1 index and Observed OTUs in the Control group were lower than those in the Normal group, indicating that the diversity of intestinal flora in rats was significantly affected by the high-fat diet. In contrast, both the LGC and HGC groups demonstrated an upward trend in their Chao1 index and Observed OTUs when compared to the Control group. The Alpha diversity of intestinal flora was directly proportional to Shannon index and inversely proportional to the Simpson index. As shown in Figure 5D,E, the Shannon index of the HGC group exhibited a higher value compared to that of the Control group, while both the LGC and HGC groups demonstrated a lower Simpson index in comparison to the Control group. The results suggest that the intervention of GC leads to an increase in species diversity among HFD rats, thereby altering the composition of gut microbiota.

As depicted in Figure 5F, at the phylum level, the gut microbiota of rats in each group primarily consisted of *Firmicutes*, *Bacteroidetes*, *Verrucomicrobia*, *Desulfobacterota*, *Proteobacteria*, *Actinobacteria*, *Fusobacteria*, and *Cyanobacteria*. Notably, *Firmicutes* and *Bacteroidetes* accounted for approximately 90% of the total composition. The relative abundance of *Proteobacteria* and *Desulfobacterota* exhibited an increase, while *Bacteroidetes* and *Verrucomicrobia* demonstrated a decrease in the Control group compared to the Normal group. Compared to the Control group, the relative abundance of *Bacteroidetes*, *Verrucomicrobia*, and *Actinobacteria* increased in the LGC and HGC groups. The relative abundance of *Firmicutes*, *Desulfobacterota*, and *Proteobacteria* was reduced. As shown in Figure 5G, the *Firmicutes*/*Bacteroidetes* ratio in the Control group exhibited a significant increase, whereas both LGC and HGC interventions led to a substantial reduction in the ratio (*p* < 0.01).

As depicted in Figure 5H, at the genus level, compared to the Normal group, the relative abundance of *Akkermansia*, *Bacteroides*, *Muribaculaceae*, *Lactobacillus*, and *Ligilactobacillus* in the Control group decreased. Conversely, the relative abundance of *Clostridiales*, *Clostridium*, and *Rikenellaceae_RC9_gut_group* increased. Compared to the Control group, after GC intervention, the relative abundance of *Akkermansia*, *Bacteroides*, and *Lactobacillus* increased significantly, while *Muribaculaceae* and *Rikenellaceae_RC9_gut_group* decreased significantly. The above results indicated that GC significantly restored the changes in the gut microbiota in HFD rats.

LEfSe (Linear discriminant analysis Effect Size) analysis was used to find the Species Biomarker with a significant difference in abundance between groups. From the LEfSe analysis results, we found that the main contributors (LDA score > 4.0) differed between the different groups, as shown in Figure 5I,J. HFD-induced communities were dominated by *Prevotellaceae*. The LGC and HGC communities were dominated by *Akkermansia*, *Rikenellaceae_RC9_gut_group*, *Lactobacillaceae*, and *Clostridiales.* The community of the HGC group was dominated by three OTUs of *Akkermansia*. The HGC group demonstrated a significantly higher proportion of *Akkermansia*, which is worth noting.

### 3.5. Effect of GC on TLR4/Myd88/NF-κB Signaling Pathway in Intestinal Tissues of HFD Rats

**Figure 6:**
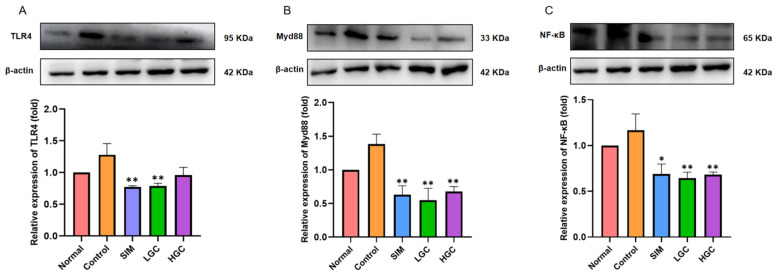
The intestinal barrier dysfunction is ameliorated by GC via the TLR4/Myd88/NF-κB signaling pathway. The protein expression levels of (**A**) TLR4, (**B**) Myd88, and (**C**) NF-κB were determined by Western Blot. SIM, LGC, HGC vs. Control, * *p* < 0.05, ** *p* < 0.01.

The structural alterations in the intestinal microbiota can lead to an imbalance in bacterial dynamics, thereby augmenting the abundance of Gram-negative bacillus and consequent endotoxin production. Elevated levels of endotoxin can bind to TLR4, initiating a molecular signaling cascade via the Myd88 pathway, thereby activating the NF-κB pathway and inducing the release of cytokines that compromise intestinal barrier integrity. This subsequently leads to bacterial endotoxin leakage into the portal circulation, triggering inflammation and oxidative stress, ultimately resulting in perturbed glucose and lipid metabolism. Western Blot analysis revealed (Figure 6) that the protein expression levels of TLR4, Myd88, and NF-κB in the intestine of HFD rats were significantly upregulated by 1.28-fold, 1.38-fold, and 1.17-fold, respectively, compared to those in the Normal group. The expression level of the TLR4 protein was significantly downregulated in the LGC group (*p* < 0.01), and had a downward trend in the HGC group, but not significantly. The expression levels of the Myd88 and NF-κB proteins in the LGC group and the HGC group were all significantly downregulated (*p* < 0.01). Interestingly, the inhibitory effect of GC on the protein expression levels of TLR4, Myd88, and NF-κB was similar to or even better than that of the SIM group. The above findings substantiate that the protective mechanism of GC on the intestinal barrier is associated with the suppression of NF-κB activation via the TLR4/Myd88 axis.

### 3.6. Correlation Analysis

**Figure 7:**
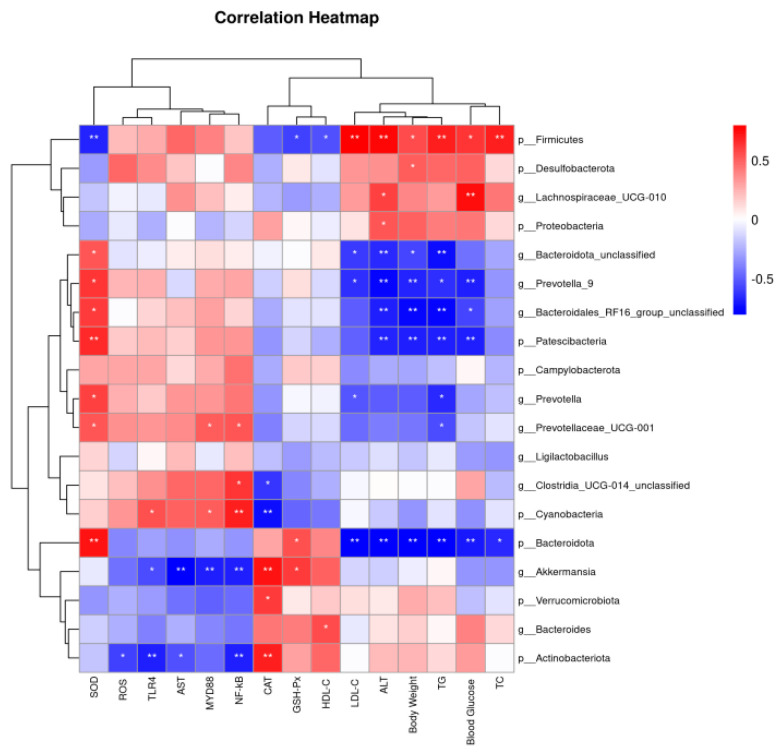
Heatmap of correlation analysis between representative gut microbiota and biochemical parameters. * *p* < 0.05, ** *p* < 0.01.

We employed Spearman analysis to examine the correlation between representative gut microbiota and biochemical parameters (physical and chemical indexes, oxidative stress levels, and related protein expression levels) (Figure 7). *Firmicutes* exhibited significant positive correlations with Blood Glucose, TC, TG, LDL-C, and ALT (*p* < 0.05 or *p* < 0.01), while showing significant negative associations with GSH-Px and SOD (*p* < 0.05 or *p* < 0.01). *Bacteroidetes* exhibited significant positive correlations with SOD (*p* < 0.01), while showing significant negative associations with Blood Glucose, Body Weight, TC, TG, ALT, and LDL-C (*p* < 0.05 or *p* < 0.01). *Bacteroides* was positively correlated with HDL-C and SOD (*p* < 0.05). *Akkermansia* was positively correlated with CAT and GSH-Px (*p* < 0.05 or *p* <0.01), and negatively correlated with AST, TLR4, Myd88, and NF-κB (*p* < 0.05 or *p* < 0.01). *Cyanobacteria* was positively correlated with TLR4, Myd88, and NF-κB (*p* < 0.05 or *p* < 0.01), and *Verrucomicrobiota* was positively correlated with CAT (*p* < 0.01). *Prevotella* was significantly negatively correlated with TG, LDL-C, and ALT (*p* < 0.05 or *p* < 0.01), *Patescibacteria* was significantly negatively correlated with Blood Glucose, Body Weight, and TG (*p* < 0.01), and *Actinobacteriota* was negatively correlated with TLR4 and NF-κB (*p* < 0.01). 

## 4. Discussion

In recent years, the research on the mechanism of Traditional Chinese Medicine (TCM) in preventing and treating glycolipid metabolism disorders has gradually transitioned from macroscopic differentiation to microscopic differentiation, and increasing evidence suggests that TCM possesses the advantages of targeting multiple pathways and targets [15]. *Gardenia jasminoides* Ellis is a kind of TCM, and its main active ingredient is crocin. Limited research has been conducted on the impact and molecular mechanism of the crude extract of crocin from *Gardenia jasminoides* Ellis (GC) in relation to glycolipid metabolism disorders. Firstly, we found that GC could significantly inhibit α-amylase and α-glycosidase activities, demonstrating its potent glucose-lowering efficacy in vitro. Secondly, in HFD rats, we found that GC could delay body weight gain, reduce blood glucose, and significantly improve lipid levels and oxidative stress in the liver. Finally, we observed that GC could significantly improve the diversity of gut microbiota, promote the proliferation of beneficial bacteria, inhibit the growth of harmful bacteria, and improve the intestinal barrier through inhibiting the TLR4/Myd88/NF-κB signaling pathway.

α-amylase and α-glucosidase play pivotal roles in the hydrolysis of polysaccharides, and inhibiting these enzymes can effectively mitigate postprandial hyperglycemia levels [16]. Currently, the drugs commonly employed for glycemic control in diabetic patients by inhibiting α-amylase and α-glucosidase, such as acarbose, exhibit significant toxic side effects [17]. Consequently, researchers are increasingly inclined towards identifying natural α-amylase and α-glucosidase inhibitors from plants that are both efficacious and devoid of toxicity. The results of the present study showed that GC significantly inhibited α-amylase and α-glucosidase compared with acarbose. Molecular docking results showed that GC had a strong binding ability to α-amylase and α-glucosidase. GC, being a carotenoid, aligns with previous research indicating the inhibitory effects of carotenoids on α-amylase and α-glucosidase. Wang et al. identified seven major carotenoids from freshwater alga Oedogonium intermedium by HPLC-PDA-APCI-IT-TOF-MS, namely neoxanthin, 9′-cis-neoxanthin, loroxanthin, violaxanthin, lutein, α-carotene, and β-carotene, and found that they had a strong inhibitory effect on α-amylase, α-glucosidase, pancreatic lipase, and other metabolic enzymes [18]. Gopal et al. found that lactucaxanthin (Lxn) in lettuce (*Lactuca sativa*) significantly inhibited (*p* < 0.05) the activity of α-amylase and α-glucosidase, and Lxn has a lower binding energy (−6.05 and −6.34 kcal mol^−1^) with α-amylase and α-glucosidase compared to their synthetic inhibitors, acarbose (−0.21 kcal mol^−1^) [19]. Our study provides a theoretical basis for the development of antidiabetic drugs without toxic side effects.

Glycolipid metabolism disorders usually manifest as increased blood glucose, significantly increased TG, TC, and LDL-C, and decreased HDL-C. Oxidative stress plays a pivotal role in the pathogenesis of diabetes and its associated complications. In terms of disease development, the excessive accumulation of adipose tissue can trigger the onset of diabetes by inducing oxidative stress damage in the liver, while an overwhelming presence of free radicals can disrupt normal glucose homeostasis [20]. Indicators are usually manifested as ALT, AST, and ROS significantly increased, and SOD, CAT, and GSH-Px significantly decreased. In this study, we observed that GC exhibited significant hypoglycemic effects, as well as pronounced reductions in TG, TC, and LDL-C levels, while concurrently increasing HDL-C levels; these findings were comparable to those of SIM. In terms of liver injury and oxidative stress, GC could significantly reduce the levels of ALT, AST, and ROS, and significantly increase the levels of CAT and GSH-Px. There are few studies on the regulation of crocin on glycolipid metabolism disorders, but some papers have found that crocin can alleviate liver oxidative stress injury, which is consistent with our results. Yaribeygi et al. found that the administration of crocin enhanced the activity of the antioxidant defense system by upregulating the levels of SOD and CAT enzymes, thereby ameliorating oxidative damage through a reduction in nitrate content and MDA production in hepatic tissues [21]. Sun et al. found that crocin could increase SOD, CAT, and GPx enzyme activities, and significantly reduce the levels of serum AST and ALT in the livers of Cisplatin-treated mice [22]. El-Beshbishy et al. found that there is an evident increase in the mRNA levels of SOD and CAT in the liver of BeCl2-intoxicated rats treated with crocin [23]. 

In recent years, the gut microbiota has been extensively studied in obesity and obesity-related diseases such as hyperglycemia [24], hyperlipidemia [25], hyperuricemia [26], and cardiovascular disease [27], etc.; specifically, metabolic syndrome associated with glucose and lipid metabolism warrants particular attention. The colonic microbiota primarily consist of anaerobic bacteria, encompassing a diverse array of bacterial species predominantly affiliated with *Firmicutes*, *Bacteroidetes*, *Actinobacteria*, *Proteobacteria*, and *Verrucomicrobia* [28]. Maintaining the diversity of gut microbiota is crucial for host health, and a decrease in diversity serves as a significant indicator of microbial imbalance within the gut [29]. 

At the phylum level, the levels of *Firmicutes*, *Verrucomicrobia*, *Desulfobacterota*, and *Proteobacteria* increased, while *Bacteroidetes* and *Actinobacteria* decreased, which would promote the development of abnormal glucose and lipid metabolism [30]. In our study, the levels of *Proteobacteria* and *Desulfobacterota* increased and *Bacteroidetes* decreased in the Control group, indicating that HFD caused gut microbiota diversity disorder, which is consistent with previous studies. The current study found that GC could increase the relative abundance of *Bacteroidetes* and *Actinobacteria* and decrease the relative abundance of *Firmicutes*, *Desulfobacterota*, and *Proteobacteria*, which indicate that GC can improve the diversity of gut microbiota in rats with HFD at the phylum level. *Firmicutes*, predominantly Gram-positive bacteria, play a pivotal role in host nutrition and metabolism by synthesizing short-chain fatty acids (SCFAs) [31]. *Bacteroides* are partly capable of dissociating and dehydrating primary bile acids and converting them to secondary bile acids in the colon, and are additionally involved in the release of toxic products during protein breakdown [32]. The presence of elevated *Firmicutes*/*Bacteroides* (F/B) ratios is considered indicative of dysbiosis and frequently associated with obesity and metabolic disorders, which may be linked to enhanced energy extraction from dietary sources, increased fat deposition and lipogenesis, as well as impaired insulin sensitivity [33]. Previous studies have found that the F/B ratio of the obese group is higher than that of the normal group, and the F/B ratio of the obese group decreases with weight loss after dietary intervention [34]. In our study, we found that GC could significantly reduce the F/B value in rats with HFD, indicating that GC could interfere with abnormal glucose and lipid metabolism by affecting F/B.

In our study, at the genus level, the relative abundance of *Akkermansia*, *Bacteroides*, and *Lactobacillus* increased significantly, while *Muribaculaceae* and *Rikenellaceae_RC9_gut_group* decreased significantly in the LGC and HGC groups, compared to the Control group. *Akkermansia* is a Gram-negative anaerobic bacterium that colonizes the intestinal mucosa of humans and rodents, specializing in degrading the mucus layer. Metagenomic data have revealed an inverse association between *Akkermansia* abundance and diseases such as inflammation, obesity, and diabetes [35]. Previous studies revealed a reduction in the abundance of *Akkermansia* in mice exhibiting obesity and type II diabetes [36]. Our study revealed that GC exerts a significant stimulatory effect on *Akkermansia* proliferation, thereby establishing a scientific foundation for the utilization of GC as a prebiotic agent and the targeting of *Akkermansia* to attenuate HFD-induced glycolipid metabolism disorder. Moreover, *Bacteroides* and *Lactobacillus*, renowned probiotics, exhibit robust antioxidant properties [37], modulate glucose and lipid absorption and metabolism pathways [38], mitigate inflammation [39], and regulate the gut microbiota [40]. The above findings suggest that GC exhibits the potential to enhance the proliferation of probiotics; however, limited research has investigated the impact of GC on intestinal microbiota and its targeted modulation on probiotics for ameliorating abnormal glucose and lipid metabolism. Our study provides a scientific basis for considering GC as a prebiotic.

The increased permeability of the intestinal barrier is a characteristic manifestation of abnormal glucose and lipid metabolism, and this heightened permeability facilitates the translocation of microbial metabolites from the intestines into the bloodstream, thereby potentially inducing metabolic endotoxemia [41]. Meanwhile, intestinal barrier permeability is frequently accompanied by intestinal inflammation, and both local and systemic inflammation can exacerbate the disruption of the intestinal milieu. Therefore, modulating inflammatory responses represents a pivotal strategy for attenuating intestinal barrier dysfunction [42]. Toll-like receptors (TLRs), a crucial family of pattern recognition receptors (PRRs), play a pivotal role in the activation of the NF-κB inflammatory pathway and maintenance of intestinal homeostasis [43]. Myd88 serves as an essential adaptor protein for all TLRs, and its deletion in the gut provides partial protection against diet-induced obesity, diabetes, and inflammation [44]. Myd88 is an important adaptor molecule necessary for TLR4 and NF-κB signaling. Activated TLR4 signals through Myd88, which in turn stresses the NF-κB pathway to keep it active and recruit downstream inflammatory factors. Therefore, this study aimed to investigate the potential mechanism by which GC exerts a protective role in inflammation and intestinal barrier damage through the regulation of the TLR4-mediated NF-κB pathway. To achieve this, we employed Western Blotting to assess the expression changes in proteins involved in the TLR4/Myd88/NF-κB signaling pathway, aiming to elucidate how GC alleviates intestinal barrier damage and subsequently modulates abnormal glucose and lipid metabolism. The present study demonstrates that GC effectively suppresses the activation of the TLR4/Myd88/NF-κB signaling pathway in the intestinal tissue of rats with HFD, thereby suggesting its potential to mitigate intestinal barrier damage and inflammation associated with HFD. We must admit that this study has some limitations. In the future, we will explore the effects of GC on the inflammation of intestinal cells and liver cells at the cellular level, as well as the effects of GC targeting intestinal inflammation, bile acid metabolism, and liver inflammation, and systematically clarify the molecular mechanism of GC targeting the gut–liver axis to interfere with abnormal glucose and lipid metabolism. It is imperative to acknowledge the limitations of this study. In future investigations, we will delve into the cellular-level effects of GC on inflammation in intestinal and liver cells, as well as its impact on targeting intestinal inflammation, bile acid metabolism, and liver inflammation, and to systematically elucidate the molecular mechanism by which GC targets the gut–liver axis to attenuate glycolipid metabolism disorder. In conclusion, this study provides a new idea for the prevention and treatment of metabolic diseases by targeting GC to regulate the intestinal barrier.

## 5. Conclusions

**Figure 8:**
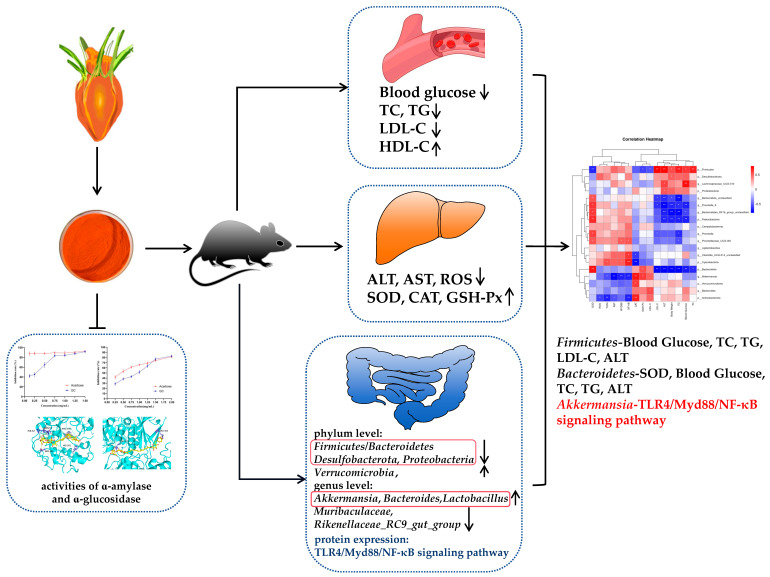
GC attenuates high-fat diet-induced glycolipid metabolism disorder in rats by targeting gut microbiota and TLR4/Myd88/NF-κB pathway. * *p* < 0.05, ** *p* < 0.01.

In conclusion, as shown in Figure 8, the present study demonstrates that GC exhibits inhibitory effects on amylase activity in vitro, suggesting its potential as a hypoglycemic agent. At the animal level, GC could significantly reduce the blood glucose and lipid levels, and improve liver oxidative stress injury in HFD rats. In addition, GC could regulate the composition of gut microbiota, reduce the *Firmicutes*/*Bacteroidetes* ratio, and promote the proliferation of probiotics such as *Akkermansia*, *Bacteroides*, and *Lactobacillus*. Moreover, the administration of GC demonstrated a significant inhibitory effect on the TLR4/Myd88/NF-κB signaling pathway, thereby attenuating intestinal barrier injury and inflammation. Finally, correlation analysis revealed a surprising finding that GC effectively targets the *Akkermansia*-TLR4/Myd88/NF-κB pathway axis to ameliorate glycolipid metabolism disorders. This study provides an innovative theoretical basis for GC as a precision nutrition dietary intervention product to target and alleviate glycolipid metabolism disorder.

## Data Availability

The data used to support the findings of this study are available from the corresponding author upon request.

## Supplementary Materials

The following supporting information can be downloaded at: https://www.mdpi.com/article/10.3390/antiox13030293/s1, Table S1: High-fat diet formulation.
