# Supplementary material for: Extract of Gardenia jasminoides Ellis Attenuates High-Fat Diet-Induced Glycolipid Metabolism Disorder in Rats by Targeting Gut Microbiota and TLR4/Myd88/NF-κB Pathway
Source: Antioxidants (Basel). 2024 Feb 28;13(3):293. doi: 10.3390/antiox13030293 (PMC10967366; doi:10.3390/antiox13030293)
Supplement: Supplementary file 1 [file antioxidants-13-00293-s001.zip › antioxidants-2873808-supplementary.pdf]

Table S1. High-fat diet formulation.

Raw Materials	Content (g/kg)
Casein	267
Maltodextrin	157
Sucrose	89
Soybean Oil	33
Lard	301
Cellulose	67
Mineral Mix,M1020	66
Vitamin Mix,V1010	13
L-Cystine	4
Choline Bitartrate	3
TBHO	0.067
Total	1000
